# Intrastromal Injection of Bevacizumab in the Management of Corneal Neovascularization: About 25 Eyes

**DOI:** 10.1155/2016/6084270

**Published:** 2016-08-17

**Authors:** Belghmaidi Sarah, Hajji Ibtissam, Baali Mohammed, Soummane Hasna, Moutaouakil Abdeljalil

**Affiliations:** Ophthalmology Department, Mohammed VI University Hospital, 40000 Marrakech, Morocco

## Abstract

*Introduction*. Corneal neovessels are a major risk factor for corneal graft rejection, due to the loss of the immune privilege. The purpose of this study is to evaluate the effectiveness of intrastromal injection of bevacizumab in the treatment of corneal neovascularization.* Material and Methods*. This is a prospective study that included 25 eyes of 22 patients with deep corneal neovessels, treated with intrastromal injections of bevacizumab.* Results*. The average age of patients was 31 years ranging from 16 to 44 years. The causes of neovascularization were dominated by herpetic keratitis (10 cases). The evolution was marked by complete regress of neovessels in 16 patients, partial regress in 6 cases, and reduced opacity and improved visual acuity in 5 patients. No side effects were noted.* Discussion*. Short-term results demonstrated the effectiveness of intrastromal injection of bevacizumab in the treatment of corneal neovessels. It may be an option or a complement to other useful treatments in stabilizing or improving vision.* Conclusion*. Bevacizumab is an effective additional treatment for the improvement of corneal transplants prognosis with preoperative corneal neovascularization.

## 1. Introduction

Corneal Neovascularization is defined as the occurrence of neovessels developed from preexisting vascular structures in the limbus, invading areas of the cornea initially not vascularized [1]. The avascular character results from a balance between angiogenic and antiangiogenic factors. In pathological conditions such as inflammatory, infectious, degenerative, or traumatic ones, this homeostasis can be broken and can be responsible for the occurrence of corneal neovessels [2].

Several therapeutic modalities have been proposed: steroids, physical therapy by superficial keratectomy or argon laser, cauterization [3], and local [4] and subconjunctival application of anti-VEGF (bevacizumab, ranibizumab); but there is so far no consensus in the therapeutic management of corneal neovessels.

The aim of our study is to report the efficiency and safety of bevacizumab in intrastromal injection in the treatment of corneal neovascularization.

## 2. Material and Methods

This is a prospective, noncomparative study, conducted at the University Hospital Mohammed VI of Marrakech, between September 2013 and December 2015, and it included 25 eyes of 23 patients with deep predescemetic corneal neovessels who received intrastromal injections of bevacizumab.

All patients underwent an eye examination with a measurement of best corrected visual acuity, examination at the slit lamp with photographs of the cornea, intraocular pressure measurement, and examination of the eye fundus with an ocular ultrasound in the absence of a refractive medium of the eye.

The severity was assessed using a clinical grading scale; this method aims to analyze directly photographs of the cornea (Table 1). Patients had to have a minimum setback of six months to be included in the study.

All patients received initially a topical corticosteroid to reduce their diameter and act on their effects (edema and inflammation). The injections were performed using an operating microscope. The bevacizumab at 25 mg/mL was prepared sterilely into a syringe with a needle of 30 gauge. An injection of about 0.01 mL was performed under topical anesthesia with instillation of oxybuprocaine hydrochloride 0.4%. The product was administered in the corneal stroma next to neovessels; the exact location depends on the location of the neovessels compared to the limbus, their number, and their extension according to the quadrants. All affected quadrants should be treated.

The intrastromal injections were spaced by a month and were continued until the stagnation of the regression of neovascularization. Patients received two injections at least before concluding an ineffective treatment. Patients who received intrastromal injections of bevacizumab were monitored at day 7, day 30, and day 120. Patients received blood pressure test, visual acuity, intraocular pressure measurement, and the slit lamp exam with photographs. The photographs on photographic slit lamp were taken in the inclusion visit then monthly until the end of the treatment. The primary evaluation criterion was the corneal surface covered with neovessels before and after treatment. The corneal surface occupied by neovessels was studied on the photos. The analysis was performed by clinical grading scale and Photoshop software (Adobe Systems, Inc., San Jose, California, United States).

The second criterion for efficacy was the variation in visual acuity before and after treatment. Safety was studied by collecting local and general side effects occurring during treatment.

## 3. Results

Twenty-five eyes of twenty-three patients (15 men and 8 women) with nine months of setback after the beginning of bevacizumab therapy were included in the study. The average age of patients at the time of treatment with bevacizumab was 31 years (between 16 and 44 years). The etiologies of corneal neovascularization were scars of herpetic keratitis in 10 patients (43.4%), ocular trauma in 6 patients (26.1%), postinfectious keratitis in 3 patients (13%), chemical burns in 2 patients (8.7%), and toxic epidermal necrolysis in 2 patients (8.7%).

All patients received topical corticosteroids. 10 patients received 3 intrastromal injections of bevacizumab, 9 received four injections, and 4 had 5 injections.

**Figure 1:**
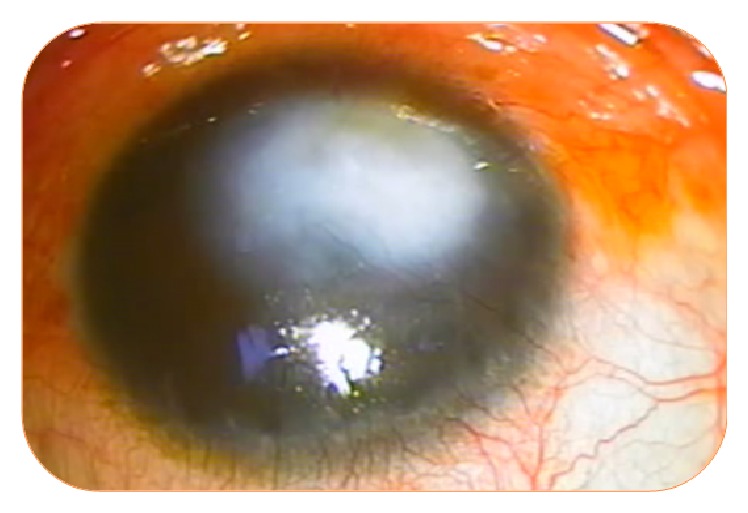
Deep neovascularization in a patient with posttraumatic opacity.

**Figure 2:**
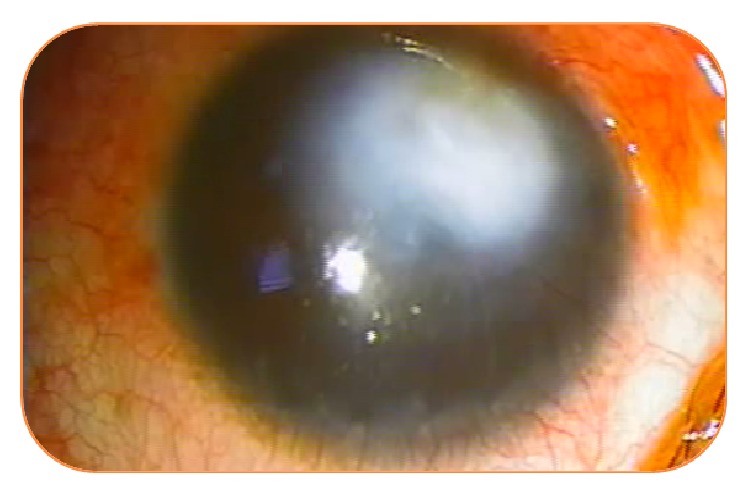
Evolution of neovascularization after 2 intrastromal injections of bevacizumab.

**Figure 3:**
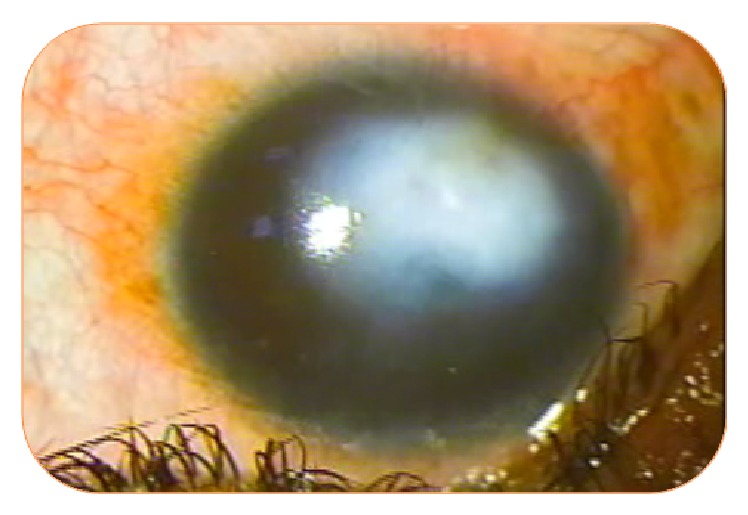
Disappearance of neovascularization after 4 injections in the same patient.

**Figure 4:**
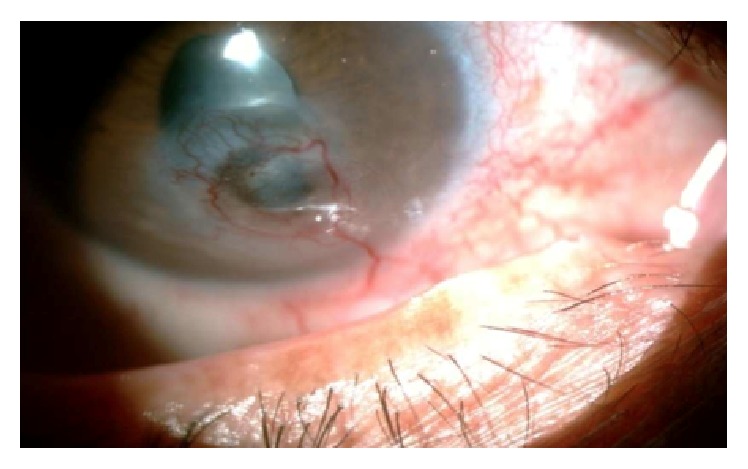
Corneal neovascularization in a patient with herpetic keratitis.

**Figure 5:**
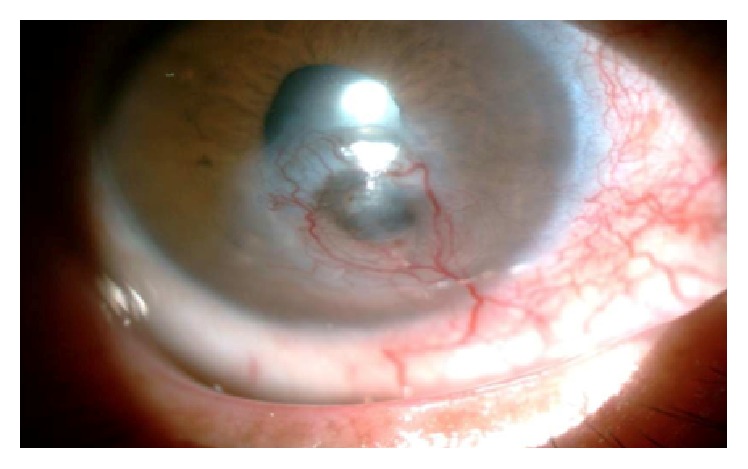
No response after three injections of bevacizumab.

The evolution was marked by the total regress in 15 patients (Figures 1, 2, and 3), partial in 5. Three patients did not respond to treatment (Figures 4 and 5). These two nonresponding patients had scars of herpetic keratitis.

Overall, the percentage of corneal neovascularization is reduced by 43 ± 19% (between 14 and 70%) at 18 ± 16% (between 0 and 39%) in Day 120 (*p* < 0.001).

Later on, 9 patients had a corneal graft with mean setback of 15 months. Intraoperatively, 6 patients received subconjunctival corticoids injections, and 2 were injected with subconjunctival bevacizumab.

Postoperatively, there was recurrence of neovessels at the collar of the receiver in 3 patients, which was well controlled under topical corticosteroids, and a backlash in 3 patients. There was also a recurrence of neovascularization in 2 patients after an average of five months. No cases of hypertension or systemic complications were noted.

## 4. Discussion

Our study seems to suggest the efficacy of bevacizumab in the treatment of deep corneal neovascularization. No local or general complications were observed.

The development of corneal neovessels occurs in several phases: a perivascular latency phase, a second active neovascularization, and finally a maturation phase of new vessels [5]. The first two phases will be a potential target for angioregressive therapy by blocking angiogenic factors [6].

Classical treatment modalities include corticoids, nonsteroidal anti-inflammatory drugs, laser photocoagulation, and reconstruction of the ocular surface. However, these treatments have demonstrated limited therapeutic effect with considerable complications [7–10].

The development of inhibitors of VEGF, a humanized monoclonal antibody bound to isoforms of VEGF-A [11], introduced a new perspective in the treatment of various ophthalmic disorders, including damage to the ocular surface such as corneal neovascularization.

The efficiency of bevacizumab in intrastromal injection has been reported; the dose of 25 mg/mL appears to be effective and well tolerated in several studies [12–15] (Table 2). The vessels had a complete response in the majority of cases. No local or general complications were reported. Results are comparable to those found in our series.

According to our study, the effectiveness varies between patients. Several hypotheses can be made to explain this variability in response to bevacizumab as the different etiologies of neovascularization are involved: the extension of the neovascularization, the state of limbo, and the delay between the appearance of neovessels and the beginning of treatment.

The improvement in visual acuity was observed partially in only two patients. Indeed, in these patients, the decline was not only due to corneal neovascularization, but also due to corneal scars.

Compared to other forms of administration, the intrastromal injection allows greater exposure to bevacizumab and delivery of a known concentration of the drug. The penetration of the topical form of this drug may be limited by an intact epithelium because of the high molecular weight of bevacizumab [16]. The intrastromal administration also ensures less risk of failure due to lack of patient compliance. Patients may sometimes forget to install the medication at home, when using a topical preparation. The subconjunctival injection of bevacizumab allows better diffusion compared to topical instillation; several studies have shown its effectiveness in the treatment of superficial and average depth neovessels [17, 18], and others have proved that subconjunctival injection of bevacizumab allows only partial reduction of deep neovessels [19–21].

A recent study showed that the subconjunctival injection has no effect on mature neovessels; it is quite effective in the active phase of the process of angiogenesis [22]. Ahn et al. used a ranibizumab having a lower molecular weight than subconjunctival topical instillation and found satisfactory results [23].

The intrastromal injection of bevacizumab increases the concentration and duration of exposure; in addition, it is also very effective in mature neovessels [13, 14]. Our study has proved its efficacy and safety in deep neovascularization.

## 5. Conclusion

The intrastromal injection of bevacizumab might be a useful option in the management of corneal neovascularization. Our study seems to show its efficacy and tolerance. However, further studies are needed to determine the optimum dosage and to define the indications, the frequency, and risk factors for developing possible side effects.

## Figures and Tables

**Table 1:** Clinical grading method of the corneal neovascularization in the degree of invasion of new blood vessels in the central cornea.

Gradation of corneal neovascularization	Score
Grade 0: <0.5 mm	0
Grade 1: >0.5 to 2 mm	1
Grade 2: >1 to 2 mm	2
Grade 3: >2 to 3 mm	3
Grade 4: >3 mm	4

**Table 2:** Deep neovessels' response to VEGF according to studies.

Series	Number of cases	Molecule	Follow-up period	Response
Vieira et al. 2012 [12]	6 cases	Bevacizumab	6 months	Total reduction: 4 Failure: 1
Yeung et al. 2011 [16]	12 cases	Bevacizumab	6 months	Complete reduction
Hashemian et al. 2011 [13]	1 case DALK	Bevacizumab	6 months	Complete reduction
Ahn et al. 2014 [23]	1 case	Ranibizumab	4 months	Partial reduction
